# Can Elevated Air [CO_2_] Conditions Mitigate the Predicted Warming Impact on the Quality of Coffee Bean?

**DOI:** 10.3389/fpls.2018.00287

**Published:** 2018-03-06

**Authors:** José C. Ramalho, Isabel P. Pais, António E. Leitão, Mauro Guerra, Fernando H. Reboredo, Cristina M. Máguas, Maria L. Carvalho, Paula Scotti-Campos, Ana I. Ribeiro-Barros, Fernando J. C. Lidon, Fábio M. DaMatta

**Affiliations:** ^1^Plant Stress & Biodiversity Group, Linking Landscape, Environment, Agriculture and Food Unit (LEAF), Departamento de Recursos Naturais, Ambiente e Território, Instituto Superior de Agronomia, Universidade de Lisboa, Oeiras, Portugal; ^2^Departamento de Ciências da Terra (GeoBioTec), Faculdade de Ciências e Tecnologia, Universidade NOVA de Lisboa, Caparica, Portugal; ^3^Unidades de Investigação e Serviços, Biotecnologia e Recursos Genéticos, Instituto Nacional de Investigação Agrária e Veterinária, I. P., Oeiras, Portugal; ^4^Laboratório de Instrumentação, Engenharia Biomédica e Física da Radiação (LIBPhys-UNL), Departamento de Física, Faculdade de Ciências e Tecnologia, Universidade NOVA de Lisboa, Caparica, Portugal; ^5^Centre for Ecology, Evolution and Environmental Changes, Faculdade de Ciências, Universidade de Lisboa, Lisbon, Portugal; ^6^Departamento de Biologia Vegetal, Universidade Federal de Viçosa, Viçosa, Brazil

**Keywords:** coffee bean quality, climate changes, *Coffea arabica*, elevated air CO_2_, warming

## Abstract

Climate changes, mostly related to high temperature, are predicted to have major negative impacts on coffee crop yield and bean quality. Recent studies revealed that elevated air [CO_2_] mitigates the impact of heat on leaf physiology. However, the extent of the interaction between elevated air [CO_2_] and heat on coffee bean quality was never addressed. In this study, the single and combined impacts of enhanced [CO_2_] and temperature in beans of *Coffea arabica* cv. Icatu were evaluated. Plants were grown at 380 or 700 μL CO_2_ L^-1^ air, and then submitted to a gradual temperature rise from 25°C up to 40°C during ca. 4 months. Fruits were harvested at 25°C, and in the ranges of 30–35 or 36–40°C, and bean physical and chemical attributes with potential implications on quality were then examined. These included: color, phenolic content, soluble solids, chlorogenic, caffeic and *p*-coumaric acids, caffeine, trigonelline, lipids, and minerals. Most of these parameters were mainly affected by temperature (although without a strong negative impact on bean quality), and only marginally, if at all, by elevated [CO_2_]. However, the [CO_2_] vs. temperature interaction strongly attenuated some of the negative impacts promoted by heat (e.g., total chlorogenic acids), thus maintaining the bean characteristics closer to those obtained under adequate temperature conditions (e.g., soluble solids, caffeic and *p*-coumaric acids, trigonelline, chroma, Hue angle, and color index), and increasing desirable features (acidity). Fatty acid and mineral pools remained quite stable, with only few modifications due to elevated air [CO_2_] (e.g., phosphorous) and/or heat. In conclusion, exposure to high temperature in the last stages of fruit maturation did not strongly depreciate bean quality, under the conditions of unrestricted water supply and moderate irradiance. Furthermore, the superimposition of elevated air [CO_2_] contributed to preserve bean quality by modifying and mitigating the heat impact on physical and chemical traits of coffee beans, which is clearly relevant in a context of predicted climate change and global warming scenarios.

## Introduction

Coffee is one of the most globally traded agricultural commodities, and constitutes the social and economic basis of many tropical developing countries, with the livelihoods of 25 million smallholder farmers depending on this crop (Waller et al., 2007). In addition, approximately 125 million people worldwide are part of the entire coffee chain of value (Osório, 2002; Pendergrast, 2010). Among the 125 *Coffea* species (Krishnan et al., 2013), *C. arabica* L. (Arabica coffee) and *C. canephora* Pierre ex A. Froehner (Robusta coffee) account together for approximately 99% of world coffee production. In the last decades world coffee bean yields increased steadily, being consistently near or above 9 million tons in the last years (International Coffee Organization [ICO], 2017), with an income of ca. US$ 173.000 million for the entire coffee chain of value (International Coffee Organization [ICO], 2014).

The coffee flavor and aroma generated during the bean roasting process are derived from chemical precursors present in the green bean. CGA (with 5-CQA constituting the major component, and its isomers 3- and 4-CQA), caffeine, trigonelline, lipids, and sucrose are among the major chemical components of coffee beans. Their contents in the green bean are closely related to the perceived quality of coffee beverage, that is, to taste, aroma, acidity, bitterness, and astringency (Koshiro et al., 2006; Farah, 2009; Barbosa et al., 2012; Ribeiro et al., 2014). CGAs have antioxidant properties and are also determinant to the final acidity, astringency, and bitterness of the beverage (Koshiro et al., 2007; Farah, 2009). Caffeine and trigonelline (another precursor of the volatile compounds) are major nitrogenous compounds, whose contents are also closely related to the quality of coffee beverage (Farah, 2009; Santos et al., 2015). Coffee is also a rich source of another major class of phenolic compounds, the hydroxycinnamic acids that include caffeic, ferulic, and *p*-coumaric acids. Therefore, coffee beverage can contribute to reduce oxidative stress in the human body, namely through the scavenging of free radicals and chelation of transition metals (Budryn and Rachwal-Rosiak, 2013), with a wide range of positive impact on human health, although high phenolic contents in green coffee may produce an undesirable flavor during the roasting process (Farah, 2009).

The relationship between climatic parameters and agricultural production is a complex process given that the impact of environmental factors on crop growth and development varies according to the species, cultivar, and phenological phases. Crop acclimation ability in a changing environment is determinant to its productivity, but the preservation of the quality is equally crucial to economic sustainability. Adverse thermal and water availability are the key environmental factors that limit coffee potential yields (DaMatta and Ramalho, 2006; International Coffee Organization [ICO], 2009). Besides, a significant number of studies has shown that coffee bean chemical composition, quality, and aroma precursors for sensorial characteristics after roasting strongly depend on the plant genetic basis, together with the prevalent environmental conditions along the bean development (e.g., soil, shade/irradiance, altitude, temperature, and water availability), bean maturation stage at harvest, agricultural practices (e.g., fertilization and thinning), and post-harvest processing (Silva et al., 2005; Vaast et al., 2006; Geromel et al., 2008; Joët et al., 2010; Barbosa et al., 2012; Bertrand et al., 2012; Cheng et al., 2016; Bote and Jan, 2017). Therefore, considering the climate changes and global warming estimates, it is crucial to study the potential impact of future environmental conditions, particularly those associated with air temperature and [CO_2_] enhancement.

Overall, it is believed that lower temperature associated with high altitude may improve coffee bean quality (Decazy et al., 2003; Avelino et al., 2005; Joët et al., 2010). This was associated with a slower maturation process of the coffee berries, delaying sugar accumulation (Geromel et al., 2008), allowing a higher accumulation of aroma precursors (Vaast et al., 2006), and the full manifestation of all of the biochemical steps required for the development of beverage quality (Silva et al., 2005). Ultimately, this leads to beans with improved organoleptic properties that are denser and far more intense in flavor than their counterparts grown at lower altitudes (or under full sunlight) (DaMatta et al., 2012). In contrast, the prevalence of supra-optimal temperatures along the bean development period will accelerate fruit growth and maturation, which might lead to quality losses (Camargo, 1985; DaMatta and Ramalho, 2006; Santos et al., 2015). This can be related to the fact that warmer conditions favor the accumulation of volatile compounds associated with the appearance of negative flavor attributes (earthy, green) (Bertrand et al., 2012).

Depending on future greenhouse gas emission scenarios, a global temperature rise from 0.3–1.7 up to 2.6–4.8°C is expected until 2100, with large ecological and agricultural effects. This is estimated to be accompanied by an increase in air [CO_2_] from the actual 405 μL L^-1^ to a value within the range of 445–1130 μL L^-1^ (Intergovernmental Panel on Climate Change [IPCC], 2014). Based on the Intergovernmental Panel on Climate Change (IPCC) scenarios, several modeling studies, using temperature as the most relevant climatic factor, have predicted decreases in biodiversity among *C. arabica* wild populations (Davis et al., 2012), strong reductions on coffee yields (Gay et al., 2006; Bunn et al., 2015), important losses of adequate areas for coffee cultivation, and shifts toward cooler regions in altitude and latitude (Zullo et al., 2011; Magrach and Ghazoul, 2015). Additionally, coffee production has already been affected by climate changes in several coffee-growing countries, due to severe drought spells in combination with supra-optimal temperatures (Bunn et al., 2015; van der Vossen et al., 2015).

In the light of the facts described above, coffee, especially *C. arabica*, is believed to be greatly sensitive to future climatic conditions, thus compromising its sustainability (Davis et al., 2012; Craparo et al., 2015). However, it is known that the coffee plant displays an interesting resilience to environmental stresses, namely those related to water scarcity and temperature (DaMatta and Ramalho, 2006; Ramalho et al., 2014). Moreover, it was recently reported remarkable heat tolerance (up to 37°C), quite above what has been traditionally accepted (Rodrigues et al., 2016), and elevated air [CO_2_] was observed to be a key player to coffee heat tolerance given that it mitigates the negative impacts of supra-optimal temperature. These facts were associated with a higher plant vigor (Ramalho et al., 2013; Ghini et al., 2015) via the maintenance of mineral nutrients balance (Martins et al., 2014), reinforcement of several photosynthetic apparatus components and protective molecules, and, above all, preservation of a higher C-assimilation performance than under actual [CO_2_], even at temperatures as high as 42°C (Martins et al., 2014, 2016; Rodrigues et al., 2016). Additionally, very recent modeling studies related with the beneficial effect of elevated [CO_2_] suggested an absence of negative impacts on coffee bean yields under warming conditions with a greater beneficial impact at higher altitudes (Rahn et al., 2018) provided that adequate water supply is available (Verhage et al., 2017). Despite these findings, virtually nothing is known about the impact of elevated [CO_2_] and its interaction with heat on the chemical composition and quality of coffee beans, which will be of utmost importance to this crop sustainability under the projected climate change conditions, as well as because premiums are paid for superior cup quality. In this context, we aimed at addressing whether the single or combined exposure to increased air temperature and [CO_2_] affect the physical and chemical attributes of green beans, providing the first insights regarding the implications of these environmental variables on coffee quality.

## Materials and Methods

### Plant Material, Experimental Conditions, and Design

Plants 1.5-year-old from *Coffea arabica* L. cv. Icatu (an introgressed cropped variety with *C. canephora* Pierre ex A. Froehner), grown in pots (12 L), were transferred from a greenhouse (ambient [CO_2_]) into walk-in growth chambers (EHHF 10000, ARALAB, Portugal), and grown under controlled conditions of temperature (25/20°C, day/night), irradiance (ca. 700–800 μmol m^-2^ s^-1^), RH (75%), photoperiod (12 h), and either 380 μL CO_2_ L^-1^ (380 plants) or 700 μL CO_2_ L^-1^ air (700 plants) until ca. 3.5 years of age (Martins et al., 2016; Rodrigues et al., 2016). Air [CO_2_] values were kept within 380 ± 5 and 700 ± 5 μL L^-1^, controlled through an IRGA system and supplied through a CO_2_ pressurized CO_2_ bottle. Nine plants from each [CO_2_] condition were then transferred to 80 L pots, and kept for another period of 9 months under the same controlled environmental conditions described above. Subsequently, these plants were submitted to a gradual temperature increase from 25/20 up to 40/30°C along ca. 3.5 months (at an approximate rate of 1°C per week). It must be emphasized that blossoming, regardless of CO_2_ treatments, was relatively continuous. Coffee fruits (at least 40 per plant and temperature treatment) were picked only at full maturation stage at 25°C (before the onset of temperature rise), and within the range of 30–35 and 36–40°C. Therefore, these fruits were submitted to increased temperatures for the last 5–10 (30–35°C) or 11–15 weeks (36–40°C) of their maturation period. Coffee beans were then characterized with regard to their physical and chemical attributes (as described below) with potential implications on quality.

The plants were maintained without restrictions of water, nutrients (mineral nutrition provided as in Ramalho et al., 2013), or root development space.

### Physical Characterization

#### Mass, Density, and Yield of Collected Beans

Coffee bean mass (of 100 beans) and density (NP 2285, 1991), as well as bean yield (bean mass/fruit mass, %), were evaluated.

#### Color Attributes

Determination of color attributes of whole bean was performed globally following Bicho et al. (2014). Briefly, color parameters, lightness (*L*^∗^), and chromaticity (coordinates *a*^∗^ and *b*^∗^) were obtained with a Minolta CR 300 colourimeter (Minolta Corp., Ramsey, NJ, United States) coupled with a glass container for solid samples (CR-A504). Measurements were performed for illuminant D65 based on Commission Internationale de l’Éclairage (CIE) *L*^∗^*a*^∗^*b*^∗^ system. The colorimeter was first calibrated to white *Yxy* coordinates (*Y* = 93.10, *x* = 0.3161, *y* = 0.3326). *L*^∗^ measures the lightness of a color and ranges from black (0) to white (100); *a*^∗^ indicates the contribution of red or green (when its value is positive or negative, respectively); and *b*^∗^ the contribution of blue or yellow (when its value is negative or positive, respectively). The elements of perceived color lightness (*L*^∗^), H°, chroma (C, saturation), and CI were determinate from the *L*^∗^*a*^∗^*b*^∗^ coordinates. Calculation of H° (=(arctg(*b*^∗^/*a*^∗^)), in degrees, sets the kind of color (red, yellow, blue, green, etc.). *C*^∗^ (=(*a*^∗2^+*b*^∗2^)^1/2^) is a measure of color saturation or purity, were calculated following (Silveira et al., 2007), whereas CI (=(1000*a*^∗^)/(*L*^∗^*b*^∗^)) (Sdiri et al., 2017) ranges between -20 (green) and +20 (orange) with 0 representing yellow.

### Chemical Characterization

#### Titratable Acidity

The titratable acidity was assessed as described in Borém et al. (2008). Briefly, 2 g FW of ground beans was added to 50 mL of water, stirred for 1 h, and filtered. An aliquot of 5 mL was added to 50 mL of water with addition of three drops of phenolphthalein followed by titration with NaOH 0.1 N. Results were expressed in milliliters NaOH 0.1 N g^-1^ DW of coffee beans.

#### Total Phenolic Content

Total phenol content was determined using the Folin–Ciocalteu method (Singleton and Rossi, 1965). Briefly, 200 mg FW of ground beans were homogenized in 20 mL acetone (70%), maintained for 2 h in the dark with stirring, and centrifuged (10,000 × *g*, 20 min, 4°C). Afterward, 250 μL of the supernatant was added to 250 μL of the Folin–Ciocalteu reagent, 5 mL of 0.71 M Na_2_CO_3_ solution, and 7 mL of deionized water. After 1 h at room temperature in darkness, Abs_750__nm_ was measured. A standard curve using gallic acid at different concentrations (1–4 mM) was performed. Results were expressed as mg GAEs g^-1^ DW.

#### Total Soluble Solids

Determination was performed according to AOAC (1984). Briefly, 1 g of ground beans was extracted with 20 mL of water, weighed, and boiled during 5 min with stirring. After cooling the mixture was brought to original volume with water and filtered. An aliquot of 2.5 mL was taken, evaporated, and dried at 105°C until constant weight.

#### Chlorogenic, Caffeic and *p*-Coumaric Acids, Caffeine, and Trigonelline

Some compounds with major known impact on the quality of the beverage were analyzed according to Alves et al. (2006): CGAs (3-CQA, 4-CQA, 5-CQA), caffeic and *p*-coumaric acids, caffeine, and trigonelline. Briefly, 0.5 g of ground beans was extracted with 30 mL of acetonitrile/water (5:95, v/v) during 10 min at 80°C, followed by filtration (Whatman Paper No. 1). An aliquot of 5 mL was diluted until 25 mL with the extraction solution and filtered (nylon, 0.45 μm). The extracts were analyzed with a HPLC Beckman System Gold (United States) equipped with a DAD (model 168) and a solvent module (model 126), using a Spherisorb ODS2 column (Waters, United States), a mixture of acetic acid/water (5:95, v/v) as eluent A, and acetonitrile as eluent B. A gradient program was used: 5% B during 5 min, from 5 to 13% B during 5 min, 13% B during 35 min, and returned to initial conditions after 1 min. Detection was at 272 nm for trigonelline and caffeine, and 320 nm for caffeic and *p*-coumaric acids and 3-CQA, 4-CQA, and 5-CQA. The identification and quantification of all CQAs were carried out using 5-CQA standard, following Trugo and Macrae (1984), whereas standard curves were used for caffeine (0.125–1 mg mL^-1^), trigonelline (0.060–0.500 mg mL^-1^), caffeic acid and *p*-coumaric acid (0.050–0.400 mg mL^-1^).

#### Fatty Acid Composition

Total lipids were obtained as described in Bertrand et al. (2008) with some modifications. Briefly, ground beans (750 mg DW) were extracted twice with 7.5 mL of methylene chloride/methanol (2:1) at ca. 20°C (room temperature) using a vortex during 1 min. After centrifugation (2700 × *g*, 10 min, 4°C) the solvent was washed (3 mL of 0.73% NaCl solution), the lower phase was recovered and evaporated to dryness under N_2_ flow at 40°C, and dissolved in 1 mL of the extraction mixture. Fatty acids (FAs) were saponified and methylated with BF_3_-methanol, using heptadecanoic acid (C17:0) as internal standard to allow quantification. The FA methyl esters were analyzed with a gas–liquid chromatograph (Varian, CP-3380, United States), equipped with a hydrogen flame-ionization detector. Separation was performed using a fused silica capillary column (DB-Wax, J & W Scientific, United States, 0.25 mm i.d. × 30 m, 0.25 μm). Column temperature was programed to rise from 80 to 200°C at 12°C min^-1^, after 2 min at the initial temperature. Injector and detector temperatures were 200 and 250°C, respectively. Carrier gas was hydrogen with a flow rate of 1 mL min^-1^, at a split ratio of 1:100 of the sample (Scotti-Campos et al., 2014). Individual FAs were identified by comparison with known Sigma and Supelco standards. TFAs corresponded to the sum of individual FAs. The unsaturation degree of TFA was obtained through the DBI (Mazliak, 1983).

#### Mineral Content

Total nitrogen was quantified using an EuroEA 3000 Elemental Analyser (EuroVector, Milan, Italy), exactly as described elsewhere (Rodrigues et al., 2010).

All other macro-(P, K, Ca, Mg, S, Cl) and micro-(Fe, Cu, Zn, Mn, Sr, Ni) nutrients were quantified using a micro-Energy Dispersive X-Ray Fluorescence (μ-EDXRF) analytical method, based in Ramos et al. (2016), with some modifications. Coffee beans were ground and ca. 1 g of the resulting powder was pressed (10 tons, 2 min) to form a cylindrical pellet of 2 cm in diameter. Considering the morphology of the coffee bean powder and the capacity to be compacted, the preparation of pellets was performed without addition of a binder. This procedure was employed both for the coffee samples and certified reference materials used for quantification validation purposes. The pellets were then placed in the focal spot of the μ-EDXRF system (M4 Tornado^TM^, Bruker, Germany). This spectrometer consists of an air-cooled micro-focus side window Rh-anode X-ray tube, powered by a low-power HV generator. The system features a poly-capillary X-ray optics, which allowed a spot size of 25 μm at the sample. The X-ray generator was operated at 50 kV and 300 μA without the use of filters, at 20 mbar vacuum conditions, to enhance the ionization of low-Z elements, such as Mg. Detection of fluorescence radiation was performed by an energy-dispersive silicon drift detector, XFlash^TM^, with 30 mm^2^ sensitive area and energy resolution of 142 eV, for an energy of 5.9 keV (corresponding to Mn K_α_).

In order to overcome the existence of elemental hotspots due to the intrinsic heterogeneity of the biological samples, the analysis was performed in an area of 7 × 7 mm. This allowed a macroscopic averaging of the concentration within the pellet. The maps were composed by a grid of around 80,000 pixels separated by 25 μm in both axes with a measuring time of ca. 3 ms per pixel, with a total of approximately 300 s.

Elemental quantification was performed through the fundamental parameter method, implemented in the commercial built-in software ESPRIT. A set of standard reference materials (SRMs) was also quantified using the same setup in order to validate the results. The SRM used in this work comprised Bush Branches (GBW 07603), Poplar Leaves (GBW 07604), and Orchard Leaves (NBS 1571), all of which with matrices similar to the samples investigated in this work. Less than 5% discrepancies (at one standard deviation level) were found for every certified elemental concentration. In order to also account for unlikely systematic errors, the final concentrations are presented with a 7% combined uncertainty.

### Statistical Analysis

Results were analyzed using a two-way *ANOVA*, considering the single and combined effects of [CO_2_] and temperature, followed by a Tukey’s test for mean comparison using the software Statistica v12 (StatSoft Inc.). A 95% confidence level was adopted for all tests.

## Results

### Physical Characterization of Coffee Beans

The gradual temperature increase has driven most changes in bean mass, density, and yield, without significant differences between [CO_2_] conditions for almost all cases (**Table 1**). Heat promoted progressive decreases in bean mass; at the highest temperature, the values represented 55 and 61% of those found at 25°C under normal and elevated [CO_2_], respectively. Similarly, bean yield value was reduced to 74% at the highest temperature relative to the initial value in both CO_2_ conditions, although with a significant decrease already in the intermediate temperature range for the 380 plants. Apparent bean density followed an opposite trend, with a significant increase at 36–40°C, but only in the beans from 380 plants (**Table 1**). Overall, intermediate temperature promoted intermediary changes in these bean traits.

**Table 1:** Bean mass (of 100 beans), density, and yield (bean mass/fruit mass, %) of coffee beans from *C. arabica* cv. Icatu collected at 25°C, and within the ranges of 30–35 and 36–40°C (diurnal temperatures).

		Diurnal temperature at bean harvest		
Parameter	[CO_2_] (μL L^-1^)	25°C	30–35°C	36–40°C
Mass of 100 beans (g)	380	9.57 ± 1.06 aA	7.17 ± 1.00 abA	5.23 ± 1.00 bA
	700	10.31 ± 0.76 aA	8.02 ± 0.79 abA	6.38 ± 0.56 bA
Density (g mL^-1^)	380	0.686 ± 0.001 bA	0.688 ± 0.001 bA	0.754 ± 0.001 aA
	700	0.654 ± 0.001 aA	0.654 ± 0.018 aA	0.700 ± 0.011 aB
Yield (%)	380	15.99 ± 0.79 aA	12.47 ± 0.83 bA	11.91 ± 1.05 bA
	700	16.26 ± 1.11 aA	13.94 ± 1.03 abA	12.10 ± 0.78 bA

*For each parameter, the mean values ± SE (*n* = 5–6, performing two replicates of five to six plants) followed by different statistical indexes express significant differences between temperatures for the same CO_*2*_ treatment (a, b), or between CO_*2*_ treatments within each temperature (A, B). Two-way *ANOVA* depicted significant changes for temperature, but not for [CO_*2*_] conditions or the interaction for all parameters.*

**FIGURE 1:**
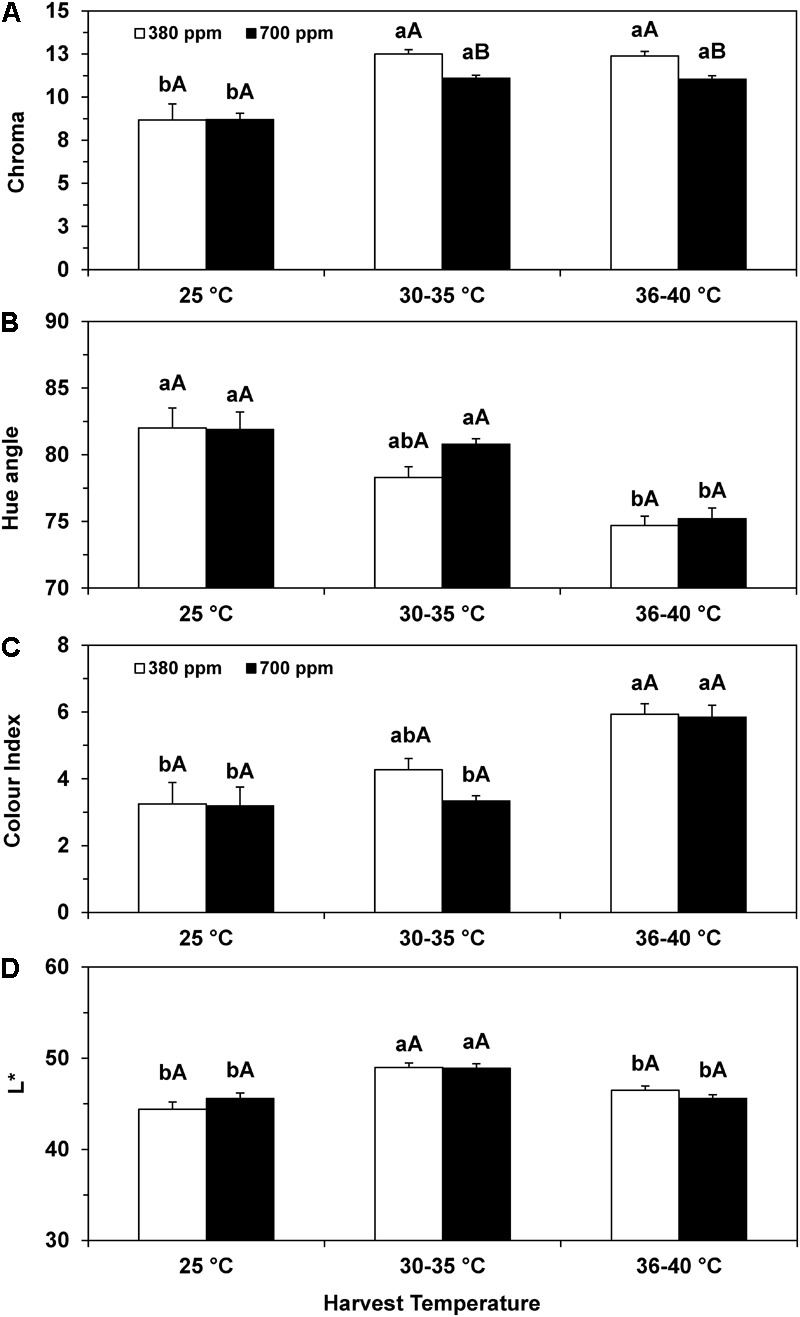
Color attributes related to chroma **(A)**, Hue angle **(B)**, color index **(C)**, and lightness (*L*^∗^) **(D)** of coffee beans from *C. arabica* cv. Icatu collected at 25°C, and within the ranges of 30–35 and 36–40°C (diurnal temperatures). For each parameter, the mean values ± SE (*n* = 9, performing two replicates of nine plants) followed by different statistical indexes express significant differences between temperatures for the same CO_2_ treatment (a, b), or between CO_2_ treatments within each temperature **(A,B)**. Two-way *ANOVA* depicted significant changes for temperature, [CO_2_] conditions, and the interaction for chroma. For the H° and CI, the *ANOVA* found significant changes for temperature, but not for [CO_2_] conditions or the interaction.

The color parameters chroma, H°, CI, and lightness (*L*^∗^) changed significantly, again more closely related to the exposure to higher temperatures during the fruit development/ripening rather than to [CO_2_] (**Figure 1**). At control temperature (25°C) there was no effect of [CO_2_] on any of these parameters. Under elevated temperatures H° was reduced and CI increased, though significantly only at the highest temperature. *L*^∗^ increased moderately (although significantly) only at the intermediate temperature range. CI was the most affected parameter, reaching increases of ca. 83% at the highest temperature range, similar for both [CO_2_]. These parameters did not respond to CO_2_. Additionally, chroma increased significantly at temperatures higher than control, but the beans from 380 plants showed significantly higher values than those from the 700 plants, due to a smaller impact in the later ones. Notably, the largest difference between [CO_2_] conditions was consistently found for H°, CI, and chroma (significantly only for the latter) at the intermediate temperature range, suggesting a lower heat impact under high [CO_2_] at this temperature.

### Chemical Characterization of Coffee Beans

Most chemical parameters and compounds were primarily altered by temperature. Total phenol content was impacted by elevated [CO_2_] at 25°C, with lower values in the beans from 700 plants. The opposite was observed at the intermediate temperature due to a strong rise of the content in these plants (**Table 2**), which was partially maintained at the highest temperature range, although without significant differences between [CO_2_]. Acidity was unaffected by [CO_2_] under control temperature (**Table 2**), but increased under the interaction of heat and [CO_2_], reaching significantly higher values in 700 plants at the highest temperature range when compared with their 380 plant counterparts. The temperature rise promoted an increase in total soluble solids, but only in beans from 380 plants (**Table 2**).

**Table 2:** Values for total phenol content, titratable acidity, total soluble solids of coffee beans from *C. arabica* cv. Icatu collected at 25°C, and within the ranges of 30–35 and 36–40°C (diurnal temperatures).

		Diurnal temperature at bean harvest		
Parameter	[CO_2_] (μL L^-1^)	25°C	30–35°C	36–40°C
Total phenol (mg GAE g^-1^ DW)	380	44.1 ± 1.8 aA	37.9 ± 1.0 bB	45.5 ± 2.8 aA
	700	35.8 ± 1.1 cB	49.4 ± 2.1 aA	42.0 ± 0.8 bA
Titratable acidity (mL NaOH 0.1 N g^-1^ DW)	380	1.95 ± 0.07 aA	2.00 ± 0.11 aA	2.09 ± 0.17 aB
	700	2.09 ± 0.10 bA	2.29 ± 0.08 abA	2.62 ± 0.07 aA
Total soluble solids (% DW)	380	34.5 ± 0.9 bA	38.4 ± 1.3 aA	37.6 ± 1.2 abA
	700	33.0 ± 1.2 aA	35.4 ± 0.5 aA	33.5 ± 0.8 aB

*For each parameter, the mean values ± SE (*n* = 5, performing two replicates of five plants) followed by different statistical indexes express significant differences between temperatures for the same CO_*2*_ treatment (a, b), or between CO_*2*_ treatments within each temperature (A, B). Two-way *ANOVA* depicted significant changes for temperature, [CO_2_], and their interaction for total phenol and acidity. For total soluble solids, significance was observed for temperature and [CO_*2*_], but not for the interaction.*

The contents of CQA isomers 3-CQA and 4-CQA (**Table 3**) strongly increased with temperature rise, irrespective of [CO_2_], reaching maximal values under the highest temperature range, with increases of 138 and 101% (4-CQA), and 299 and 228% (3-CQA) in the beans from 380 and 700 plants, respectively. In contrast, the most represented CQA isomer (5-CQA) was almost unresponsive to heat alone, and was reduced by 17% under the heat vs. [CO_2_] interaction (700 plants) at 36–40°C. Altogether, these changes modified the proportion of individual CQAs in total CQA content. Under temperature control, 5-CQA represented ca. 85% of total CQAs for both [CO_2_] treatments, decreasing to 64% at the highest temperature range, whereas 3-CQA and 4-CQA represented ca. 6–7 and 8% of total CQAs at 25°C, increasing to 20–21 and 16% at the highest temperature range, respectively. With regard to the interaction of heat and elevated air [CO_2_], only 4-CQA showed some differences, with the beans from 700 plants displaying a lower value than those from 380 ones. Still, in 380 plants the smaller 5-CQA decrease, and the strong 3- and 4-CQA rises, led to a significant increase in total CQAs at 36–40°C, compared to their control and to the 700 plants (that revealed a more stable value of total CQAs).

**Table 3:** Values for total and individual CQAs, caffeic acid, caffeine, trigonelline, and *p*-coumaric acid of coffee beans from *C. arabica* cv. Icatu collected at 25°C, and within the ranges of 30–35 and 36–40°C (diurnal temperatures).

		Diurnal temperature at bean harvest		
Parameter	[CO_2_] (μL L^-1^)	25°C	30–35°C	36–40°C
5-CQA	380	31.5 ± 1.4 aA	30.7 ± 1.5 aA	28.0 ± 1.3 aA
(mg g^-1^ DW)	700	30.6 ± 1.6 aA	30.1 ± 1.2 aA	25.4 ± 0.6 bA
4-CQA	380	2.94 ± 0.14 cA	3.99 ± 0.27 bA	7.01 ± 0.54 aA
(mg g^-1^ DW)	700	3.08 ± 0.28 cA	4.70 ± 0.22 bA	6.19 ± 0.29 aB
3-CQA	380	2.21 ± 0.13 cA	3.91 ± 0.45 bB	8.82 ± 0.13 aA
(mg g^-1^ DW)	700	2.51 ± 0.22 cA	5.06 ± 0.25 bA	8.24 ± 0.24 aA
Total CQAs	380	36.7 ± 1.4 bA	38.6 ± 1.4 abA	43.9 ± 1.3 aA
(mg g^-1^ DW)	700	36.2 ± 1.5 aA	39.9 ± 1.4 aA	39.8 ± 0.8 aB
Caffeic acid	380	0.083 ± 0.003 aA	0.073 ± 0.007 abB	0.065 ± 0.006 bA
(mg g^-1^ DW)	700	0.083 ± 0.007 aA	0.090 ± 0.004 aA	0.074 ± 0.004 aA
Caffeine	380	11.3 ± 0.6 bA	13.8 ± 0.4 aA	13.0 ± 0.6 abA
(mg g^-1^ DW)	700	11.6 ± 0.6 aA	10.2 ± 0.6 abB	9.5 ± 0.4 bB
Trigonelline	380	10.9 ± 0.7 cA	14.1 ± 0.7 bA	17.5 ± 1.2 aA
(mg g^-1^ DW)	700	10.1 ± 0.7 bA	13.7 ± 0.6 aA	14.9 ± 0.4 aB
*p*-Coumaric acid	380	0.538 ± 0.055 cA	0.964 ± 0.068 aA	0.740 ± 0.071 bA
(mg g^-1^ DW)	700	0.703 ± 0.027 aA	0.602 ± 0.029 aB	0.561 ± 0.049 aB

*For each parameter, the mean values ± SE (*n* = 5, performing two replicates of five plants) followed by different statistical indexes express significant differences between temperatures for the same CO_*2*_ treatment (a, b, c), or between CO_*2*_ treatments within each temperature (A, B). Two-way *ANOVA* depicted significant changes for temperature, [CO_*2*_], and their interaction for total CQAs, caffeine, trigonelline, and *p*-coumaric acid; significant differences were found for temperature and [CO_*2*_] on 4-CQA, whereas for 5-CQA, 3 CQA, and caffeic acid only temperature promoted significant changes.*

Caffeic acid did not change significantly along the experiment in the 700 plants, but gradually decreased at temperatures higher than the control in 380 plants, reaching significance at the 36–40°C range. Caffeine and *p*-coumaric acid levels significantly increased with rising temperature in 380 plants, especially in the 30–35°C range, whereas the temperature vs. [CO_2_] interaction promoted an opposite trend in 700 plants. This resulted in higher values of caffeine and *p*-coumaric acid in the beans from 380 plants than in 700 plants at temperatures higher than control. Trigonelline increased at 30–35°C and onward on both [CO_2_], reaching maximal increases of 61 and 48% in the 380 and 700 plants, respectively, at 36–40°C. Enhanced [CO_2_] significantly attenuated such heat impact.

Regarding the lipid profile (**Table 4**), no significant variations due to [CO_2_] and/or supra-optimal temperatures were observed in the DBI, and TFAs, which integrated the changes of individual FAs. Accordingly, the proportion of the majority of FAs was not significantly modified. Globally, C18:2 was the most represented FA (ca. 41–43%), closely followed by C16:0 (ca. 35–36%). Another two C18 FAs, C18:1 and C18:0, represented ca. 9–10 and 7–8% of TFAs, respectively. The C18:3, as well as the long carbon chain FAs (C20:0, C20:1, C22:0, C24:0) comprised <3% of TFAs. Traces of myristic (C14:0), pentadecyclic (C15:0), C16:1 acids were still detected (<0.1%). The minor representative C20:1 (<0.8%) was the only FA that changed significantly in response to [CO_2_] conditions under control temperature. With a few exceptions (C18:0, C24:0), temperature increase alone did not significantly change FAs proportion. The combined exposure of elevated air [CO_2_] and heat altered only C20:1. Therefore, FAs were remarkably stable irrespective of [CO_2_] and temperature.

**Table 4:** Changes in TFAs content (mg g^-1^ DW), FA composition (% mol), and unsaturation (DBI) of coffee beans from *C. arabica* cv. Icatu collected at 25°C, and within the ranges of 30–35 and 36–40°C (diurnal temperatures).

		Diurnal temperature at bean harvest		
Parameter	[CO_2_] (μL L^-1^)	25°C	30–35°C	36–40°C
TFA	380	58.6 ± 0.1 aA	58.2 ± 2.0 aA	53.7 ± 1.9 aA
(mg g^-1^ DW)	700	56.4 ± 2.5 aA	53.0 ± 1.5 aA	56.2 ± 1.8 aA
C16:0	380	35.2 ± 0.1 aA	35.3 ± 1.8 aA	32.6 ± 1.3 aB
(% mol)	700	35.8 ± 0.1 aA	34.8 ± 1.1 aA	36.3 ± 0.7 aA
C18:0	380	6.90 ± 0.30 bA	7.00 ± 0.40 bA	8.30 ± 0.30 aA
(% mol)	700	7.50 ± 0.40 aA	7.70 ± 0.20 aA	8.00 ± 0.20 aA
C18:1	380	9.80 ± 0.20 aA	9.50 ± 1.10 aA	10.40 ± 0.40 aA
(% mol)	700	9.80 ± 0.50 aA	9.40 ± 0.40 aA	8.90 ± 0.50 aA
C18:2	380	43.4 ± 0.1 aA	43.4 ± 0.2 aA	42.4 ± 0.7 aA
(% mol)	700	41.5 ± 0.4 aA	41.3 ± 1.1 aB	41.3 ± 0.8 aA
C18:3	380	1.00 ± 0.10 aA	0.80 ± 0.20 aA	1.10 ± 0.10 aA
(% mol)	700	1.00 ± 0.00 aA	1.10 ± 0.10 aA	1.30 ± 0.00 aA
C20:0	380	2.40 ± 0.10 aA	2.40 ± 0.00 aB	3.00 ± 0.20 aA
(% mol)	700	2.50 ± 0.10 bA	3.40 ± 0.20 aA	2.60 ± 0.30 bA
C20:1	380	0.310 ± 0.010 bB	0.550 ± 0.110 aB	0.550 ± 0.070 aA
(% mol)	700	0.570 ± 0.030 abA	0.770 ± 0.090 aA	0.460 ± 0.060 bA
C22:0	380	0.680 ± 0.100 aA	0.660 ± 0.220 aA	1.060 ± 0.030 aA
(% mol)	700	0.690 ± 0.020 aA	1.020 ± 0.070 aA	0.740 ± 0.120 aA
C24:0	380	0.210 ± 0.020 abA	0.130 ± 0.060 bA	0.270 ± 0.010 aA
(% mol)	700	0.190 ± 0.020 aA	0.230 ± 0.020 aA	0.180 ± 0.030 aA
DBI	380	2.20 ± 0.00 aA	2.20 ± 0.10 aA	2.20 ± 0.10 aA
	700	2.10 ± 0.00 aA	2.00 ± 0.10 aA	2.00 ± 0.00 aA

*For each parameter, the mean values ± SE (*n* = 4, performing two replicates of four plants) followed by different statistical indexes express significant differences between temperatures for the same CO_*2*_ treatment (a, b), or between CO_*2*_ treatments within each temperature (A, B). Although a few mean differences were observed, the two-way ANOVA only depicted significant changes [CO_*2*_] for C20:1. DBI = [%monoenes + 2 × %dienes + 3 × %trienes9/%saturated FAs] (Mazliak, 1983). Traces of C14:0, C15:0, and C16:1 (<0.1% mol) were also found.*

### Impact on Mineral Composition in the Coffee Bean

The composition of macro-(N, P, K, Ca, Mg, S, Cl) and micro-(Fe, Cu, Zn, Mn, Sr, Ni) nutrients (**Table 5**) remained mostly unaltered in response to elevated air [CO_2_] under control temperature, with the exception of P and Cl (whose levels decreased). With increasing air temperature mineral relations were maintained between [CO_2_] treatments for most elements. Notably, at supra-optimal temperatures the macronutrient contents were unresponsive in the 700 plants, whereas in 380 plants Ca and Mg increased. In contrast, there were alterations in the content of some micronutrients (e.g., decreases of Fe, Cu, Ni; increase of Sr) in the 700 plants at the highest temperature range, whereas in 380 plants only Mn and Sr contents were significantly increased.

**Table 5:** Leaf macronutrient (mg g^-1^ DW) and micronutrient (μg g^-1^ DW) contents in *C. arabica* cv. Icatu collected at 25°C, and within the ranges of 30–35 and 36–40°C (diurnal temperatures).

		Diurnal temperature at bean harvest		
Mineral	[CO_2_] (μL L^-1^)	25°C	30–35°C	36–40°C
		**Macronutrients**		

N	380	24.4 ± 1.0 aA	25.9 ± 1.5 aA	26.8 ± 1.4 aA
(mg g^-1^ DW)	700	22.5 ± 0.7 aA	23.1 ± 0.4 aA	24.2 ± 0.6 aA
P	380	2.47 ± 0.07 aA	2.43 ± 0.06 aA	2.35 ± 0.05 aA
(mg g^-1^ DW)	700	2.25 ± 0.06 aB	2.20 ± 0.04 aB	2.33 ± 0.05 aA
K	380	16.7 ± 0.5 aA	17.1 ± 0.4 aA	17.6 ± 0.2 aA
(mg g^-1^ DW)	700	16.9 ± 0.5 aA	17.7 ± 0.3 aA	17.5 ± 0.4 aA
Ca	380	2.10 ± 0.15 bA	2.75 ± 0.29 abA	3.00 ± 0.20 aA
(mg g^-1^ DW)	700	2.23 ± 0.14 aA	2.25 ± 0.22 aA	2.88 ± 0.11 aA
Mg	380	2.81 ± 0.86 bA	3.95 ± 0.28 aA	4.00 ± 0.10 aA
(mg g^-1^ DW)	700	3.45 ± 0.15 aA	3.83 ± 0.41 aA	3.83 ± 0.28 aA
S	380	2.67 ± 0.12 aA	2.83 ± 0.09 aA	2.80 ± 0.00 aA
(mg g^-1^ DW)	700	2.58 ± 0.05 aA	2.58 ± 0.03 aA	2.63 ± 0.03 aA
Cl	380	2.03 ± 0.03 aA	1.80 ± 0.15 aA	1.95 ± 0.05 aA
(mg g^-1^ DW)	700	1.78 ± 0.06 aB	1.83 ± 0.06 aA	1.90 ± 0.04 aA

		**Micronutrients**		

Fe	380	190 ± 21 aA	200 ± 34 aA	175 ± 5 aA
(μg g^-1^ DW)	700	260 ± 42 aA	168 ± 10 bA	170 ± 16 bA
Cu	380	49.3 ± 9.0 aA	46.8 ± 12.0 aA	55.5 ± 5.5 aA
(μg g^-1^ DW)	700	75.3 ± 10.5 aA	46.0 ± 5.7 abA	38.0 ± 3.5 bA
Zn	380	49.3 ± 3.8 aA	58.3 ± 9.5 aA	62.0 ± 3.0 aA
(μg g^-1^ DW)	700	64.5 ± 8.3 aA	51.3 ± 3.6 aA	45.5 ± 3.6 aA
Mn	380	39.0 ± 3.0 bA	50.5 ± 2.5 abA	54.5 ± 4.5 aA
(μg g^-1^ DW)	700	42.0 ± 1.8 aA	41.8 ± 0.5 aA	49.8 ± 1.8 aA
Sr	380	5.67 ± 0.72 bA	8.25 ± 0.89 bA	12.50 ± 1.06 aA
(μg g^-1^ DW)	700	5.25 ± 0.41 bA	5.25 ± 0.41 bB	8.50 ± 0.43 Ab
Ni	380	BDL	BDL	BDL
(μg g^-1^ DW)	700	16.33 ± 1.62 a	8.00 ± 1.53 b	6.00 ± 0.00 b

*For each parameter, the mean values ± SE (*n* = 4, performing two replicates of four plants) followed by different statistical indexes express significant differences between temperatures for the same CO_2_ treatment (a, b), or between CO_2_ treatments within each temperature (A, B). Although a few mean differences were observed, the two-way *ANOVA* depicted significant changes for [CO_2_] (P, Sr), temperature (Ca, Mg, Fe, Cu, Zn, Mn, Sr), and the interaction (Cu, Zn). BDL, below detection limit.*

## Discussion

Agricultural productivity assessments are usually carried out under field conditions, limited to the actual environmental conditions. However, potted experiments can be suitable for quality evaluation, provided that the product shows similar characteristics to those obtained under field conditions. In this work, the green beans obtained from coffee plants grown in large pots under control conditions were largely similar to those obtained under field conditions for *C. arabica* plants for most of the studied parameters (see below). Therefore, it is expected that the changes promoted in green beans by temperature increase, [CO_2_], and their interaction, would happen to a similar extent to that of the field. In this context, the major importance of our work was to contribute to unravel the effects on bean quality traits associated with the single or combined exposure to elevated [CO_2_] and supra-optimal temperatures during the fruit maturation period. In any case, whereas our data clearly suggest a role of elevated [CO_2_] on the quality of beans developed at high temperature, it must be emphasized that fruits from the plants exposed to supra-optimal temperatures initiate their development (including the fruit expansion and filling phases) at 25°C. Therefore, our results are strictly valid to the effects of supra-optimal temperatures for the last stages of fruit maturation period, when important changes occur in coffee bean chemical composition (namely related to CGAs), with impact on its quality (Bertrand et al., 2003; Montavon et al., 2003; Oestreich-Janzen, 2013).

### Coffee Bean Physical Traits

At control temperature, the apparent density values were close to those of field collected beans of *C. arabica* cultivars that show values between 0.63 and 0.68 g mL^-1^ (Molin et al., 2008; Bicho et al., 2014), although the mass of 100 beans (**Table 1**) was lower than the usual range (ca. 15–20 g) under sun or shade cultivation (Ricci et al., 2006; Bicho et al., 2014). Temperature has driven most of the observed changes regarding the bean yield, mass, and increasing apparent density, whereas elevated [CO_2_] *per se* did not alter these traits. However, the [CO_2_] vs. temperature interaction tended to attenuate the changes of these parameters, leading to values closer to the control (25°C, 380 μL CO_2_ L^-1^) in the 700 plants when compared with the 380 plants, although only density varied significantly between [CO_2_] conditions at the highest temperature range.

Chromatic characteristics of coffee beans are used to assess product quality due to its relation with the beverage, further allowing a fast, reliable, low cost, and non-destructive analysis (Farah et al., 2006; Borém et al., 2013; Bicho et al., 2014). Bean color depends on factors related to cultivars and postharvest processing (Coste, 1992), and can be modified by environmental conditions (Cheng et al., 2016). Reflectance measurements in *C. arabica* beans indicated that color intensity increased (darker) as their quality decreased, linked to phenol oxidation which changes flavor and aroma precursors (Afonso and Corrêa, 2003; Farah et al., 2006; Borém et al., 2013). The preservation of quality was also associated with the stability of bean color attributes related to the blue–green or green tones (Borém et al., 2013). Under adequate temperature, air [CO_2_] did not modify any of the studied color parameters (**Figure 1**), which presented values close to those of field-harvested *C. arabica* beans (e.g., H° – 81.5) (Bicho et al., 2014). In contrast, heat increased chroma, CI, and *L*^∗^, and reduced H° for both [CO_2_] (**Figure 1**), suggesting some degree of altered bean quality. Still, *L*^∗^ increase agreed with a lighter (closer to white) bean color at intermediate temperature range, and to similar values to those of control at the highest temperatures. Also, values of 17 for chroma, and 88 for H° were reported in non-defective *C. arabica* green beans (Mendonça et al., 2009), close to those reported for Icatu along the experiment regardless of [CO_2_]. Additionally, defective black green beans showed lowered chroma (6) and increased H° (96) (Mendonça et al., 2009), in opposition to the increased chroma and decreased H° in our samples. Thus, the heat-driven changes in color attributes suggest that oxidative processes and natural enzymatic biochemical transformation did not occur, with a concurrent maintenance of quality. Interestingly, at the intermediate temperature range, bean color attributes (except for *L*^∗^) tended to be consistently less affected in the beans from 700 plants, with marginal changes of H° and CI (relative to 25°C), and a significantly lower value for chroma, when compared to 380 plants, thus suggesting a mitigation of heat impact under elevated [CO_2_].

In summary, temperature was the most important driving force concerning the changes on the physical bean characteristics, although elevated [CO_2_] attenuated, to some extent, the heat impact (**Figure 1** and **Table 1**) on these bean quality traits.

### Coffee Bean Chemical Characteristics

Overall, the values of the studied chemical traits from beans collected under control conditions were close to those from field conditions (**Tables 2, 3**), as has been reported elsewhere. For example, field-collected beans of Icatu and other *C. arabica* cultivars showed total soluble solids and titratable acidity values between 33.2–33.8% DW and 1.98–2.38 mL NaOH 0.1 N g^-1^ DW, respectively (Lopes et al., 2000). Additionally, it were reported values (mg g^-1^ DW) within the range of 44–57 for total CQAs, 35–52 for 5-CQA, 8–16.7 for caffeine, 6–13.8 for trigonelline, and 0.01–0.21 for caffeic acid (Farah et al., 2006; Bertrand et al., 2008; Mussatto et al., 2011; Bicho et al., 2012, 2013; Kitzberger et al., 2013; Oestreich-Janzen, 2013; Babova et al., 2016). Among these parameters (**Tables 2, 3**) only total phenol content was significantly modified by the enhanced [CO_2_], whereas temperature was again the most important driver to alter chemical attributes. Temperature effect on bean traits was previously reported (e.g., Joët et al., 2010; Barbosa et al., 2012), with CGAs being dependent on mean air temperature during seed development (Joët et al., 2010). However, for the first time ever, an important interaction between temperature and [CO_2_] was also observed.

In 380 plants most compounds showed heat promoted changes, frequently in a gradual manner according to the intermediate (30–35°C) or highest (36–40°C) temperature range. This was the case of caffeic acid (decreased), total soluble solids, 3-CQA, 4-CQA, total CQAs, caffeine, trigonelline, and *p*-coumaric acid (increased), likely having potential impacts on the bean quality due to the known contribution of these compounds to coffee beverage sensorial aspects (Farah et al., 2006; Koshiro et al., 2007; Farah, 2009; Joët et al., 2010). The increase of CQAs level has an inverse association with cup quality, particularly concerning 5-CQA (Farah et al., 2006; Farah, 2009; Barbosa et al., 2012). This seems to constitute the major substrate for polyphenol oxidase in coffee, producing ortho-quinones that in turn will cause darkening of the beans and a worse quality (Mazzafera and Robinson, 2000). However, increases of total CQA associated with temperature were unrelated to the more representative 5-CQA. Instead, it relied on the strong rise of 4-CQA and 3-CQA contents, which doubled and tripled, respectively, their proportion among total CQAs. These findings fully agree with the positive correlation observed between temperature and both 3-CQA and 4-CQA, and a reverse trend in 5-CQA (the latter accumulating with altitude) under field conditions (Bertrand et al., 2006; Joët et al., 2010). This further indicates that temperature may act directly on the CGA metabolic pathway by routing toward the different isomers within the CGA metabolic pathway, as suggested by Joët et al. (2010). Since caffeine (together with CGAs) is also responsible for coffee bitterness (Joët et al., 2010; Santos et al., 2015), its increased content as temperature rose would also contribute to a poor bean quality, although Franca et al. (2005) have reported higher caffeine content for high-quality *C. arabica* beans. By opposition, a positive impact on quality could have resulted from trigonelline increase, which is a precursor of volatile compounds that contribute to the aroma and taste of roasted coffee (Barbosa et al., 2012). Such improved cup quality can be sensed even with lower increases (from 9.6 to 13.4 mg g^-1^ DW green bean, Farah et al., 2006) than those observed in our experiment (10.9–17.5 mg g^-1^ DW). Also, among the two studied hydroxycinnamic acids, caffeic and *p*-coumaric, the latter was more representative and increased with heat, reinforcing the polyphenol weight, which might contribute to a better antioxidant capacity, although also leading to a higher bitterness. Taken together, the changes driven by heat in the last stages of fruit maturation suggest some loss of quality, but to a lesser extent than that usually attributed to high temperatures under field conditions. Nonetheless, high temperatures in the field may occur with concurrent decreased water availability and high atmospheric vapor pressure deficit (compared to the controlled conditions in this study). Given that water supply *per se* might affect coffee bean quality (Vinecky et al., 2017), it is anticipated that a negative interaction between water supply and temperature could occur under field conditions, which would ultimately exacerbate impairments on coffee bean quality. If so, these facts could help explaining why we found a weaker heat impact on bean quality than that has been usually reported.

The marginal impact of elevated [CO_2_] on coffee bean quality contrasted with the enhancement of crop yields provided that adequate water supply is available (DaMatta, 2018). Moreover, enhanced [CO_2_] was found to impair the quality of food harvestable products of several crops, such as wheat, rice, barley, potato (by decreasing protein concentration), and potato (by reducing minerals content) (Taub, 2010; Dietterich et al., 2015). This was not the case in the coffee bean. In fact, the photosynthetic stimulation under elevated [CO_2_] (together with a high sink strength) improved coffee plant performance (Ramalho et al., 2013; Ghini et al., 2015) and bean yields (Ghini et al., 2015), while allowing unaltered quality (this study). These findings are in line with those observed in other species such as grapevine (Moutinho-Pereira et al., 2009), in which elevated [CO_2_] did not promote noticeable quality changes in the wine (Gonçalves et al., 2009).

Remarkably, entirely different patterns of variation (depending on the compound) were observed under the interaction of elevated air [CO_2_] vs. temperature, which were not predictable from the single effects of high temperature or [CO_2_]. In fact, high [CO_2_] attenuated, amplified, or even reverted the changes observed under the single exposure to high temperature. First, total phenol (at 30–35°C) and acidity strongly increased, the latter usually associated with beans of better aromatic quality (Bertrand et al., 2012). Second, 5-CQA and caffeine markedly decreased, possibly also reflecting a better bean quality given that higher levels of CQA, and their oxidation products, are associated with poor cup quality (Koshiro et al., 2007; Farah, 2009). Third, elevated [CO_2_] canceled (soluble solids, total CQAs, caffeic acid) or attenuated (4-CQA, trigonelline) the changes driven by heat, that is, the levels of these compounds remained closer to their initial values. Fourth, *p*-coumaric acid showed even a reversed behavior (decreased), leading to lower contents in 700 plants than in 380 plants at temperatures above control. This is in agreement with the decrease in 5-CQA, since *p*-coumaric acid is a precursor of 5-CQA (Koshiro et al., 2007). Altogether, this could be seen as a [CO_2_] positive effect on the maintenance of the actual quality standards.

Lipids are major components of *C. arabica* green beans (representing ca. 11–20% of the dry matter) (Farah, 2012; Oestreich-Janzen, 2013; Toci et al., 2013), being responsible for flavor carriers, texture, and mouthfeel in the beverage (Oestreich-Janzen, 2013; Cheng et al., 2016). Within the lipid fraction triacylglycerols represent, on average, 75% of the total lipid mass (Farah, 2012; Oestreich-Janzen, 2013), and their FAs have become chemical descriptors used to differentiate coffee varieties (Speer and Kölling-Speer, 2006). Among the most important unsaturated FAs for coffee freshness are linoleic (C18:2(*n*-6)), oleic (C18:1(*n*-9)), and linolenic (C18:3(*n*-3)) acids, which accounted for by 36–54, 7–14, and 1–2.6% of the triacylglycerol fraction, respectively (Farah, 2012; Toci et al., 2013). TFA profile from Icatu coffee bean (**Table 4**) has fallen within the expected proportion of their FAs, namely regarding C18:2, C16:0, C18:1, and C18:0, the most representative ones (Speer and Kölling-Speer, 2001; Toci et al., 2013). Additionally, C18:1 and C18:0 showed close values, which is characteristic of *C. arabica* beans (Speer and Kölling-Speer, 2001), thus reinforcing the validity of our results. In sharp contrast to most of the other parameters, the TFA contents, the proportion of individual FAs, and the DBI were largely insensitive to both [CO_2_] and temperature rises (**Table 4**), with few exceptions regarding minor FAs. Therefore, these environmental changes are not expected to have negative implications to bean quality concerning the lipid matrix, as FAs can be oxidized during storage and after roasting, decreasing beverage quality (Toci et al., 2013). These results apparently contrasted with findings that FA contents in bean depend on mean air temperature during seed development (Joët et al., 2010). This latter study used a broad climatic variation, strongly related to altitude, with the average temperature in the last 5 months preceding harvest between 14.4 and 25.3°C. However, given that this temperature range was well below that used in our experiments, direct comparisons between these studies cannot be made.

### Mineral Content in the Green Bean

Mineral nutrition can also affect bean and beverage quality, with implications to the minerals present in a cup of coffee, where it represents about 6% (Viani, 1991). In particular, N and K chemical form and amount supplied through fertilization can affect organoleptic bean quality attributes, as well as caffeine, total sugars, and acidity in *C. arabica* beans (Silva et al., 2002; Malta et al., 2003; Bote and Jan, 2017). Here, acidity (**Table 2**) and caffeine (**Table 3**) increased and decreased, respectively, in the beans from 700 plants, although without concurrent changes of N and K content (**Table 5**). In fact, green bean did not show appreciable macronutrients changes due to elevated air [CO_2_] (except for P) under control temperature (**Table 5**), with mineral values being maintained close to those observed in field evaluations (Malavolta, 1993; Covre et al., 2016). Still, knowing that P represents >80% of the mineral content present in instant coffee cups (Viani, 1991), some quality effects might arise.

With temperature rise most macro- and micronutrients presented a rising tendency at leaf level [CO_2_] (Martins et al., 2014), but not at bean level. Only a few significant differences at the highest temperature were observed when compared to beans collected at 25°C (Ca, Mg, Mn, Sr – 380 plants; Fe, Cu, Sr, Ni – 700 plants), whereas differences between air [CO_2_] conditions were found only for Sr (and probably Ni) at 42°C. Therefore, the mineral matrix can be considered quite stable under high [CO_2_] and warming, and no appreciable quality changes will be expected regarding these bean components, with the exception of P.

## Conclusion

These novel observations suggest: first, heat impacts were observed in all physical attributes, with increases in apparent density coupled with decreases in bean mass and yield. Additionally, chroma, CI, and *L*^∗^ increased, whereas H° decreased, suggesting that bean quality was not impaired. Second, supra-optimal temperatures *per se* increased 3-CQA, 4-CQA, total CQAs, caffeine, trigonelline, and *p*-coumaric acid, but reducing the content of caffeic acid, which might lead to an increase in bitterness. Third, the increase in air [CO_2_] *per se* did not significantly affect most physical and chemical parameters under adequate temperature, maintaining an overall stability of physical and chemical parameters. Fourth, a large number of parameters showed different patterns of variation under interacting conditions of elevated [CO_2_] and temperature. In some cases (e.g., H°, CI) the smaller impact promoted by the combination of [CO_2_] and heat (in comparison to heat alone) was only found at 30–35°C. However, for several other parameters the bean characteristics were kept closer to the temperature control (i.e., closer to the values observed nowadays under adequate cropping conditions), even at the highest temperature range. In fact, elevated [CO_2_] mitigated (chroma, 4-CQA, trigonelline) or even canceled (density, soluble solids, total CQAs, caffeic acid) the heat impacts on bean traits. Furthermore, it amplified (higher acidity; lower 5-CQA) or changed in the opposite direction (lower caffeine and *p*-coumaric acid) a few characteristics, likely without impairing quality, maintaining a more stable value of total CQAs. Fifth, FAs and DBI were, overall, quite stable, and largely insensitive to elevated [CO_2_] (except C20:1) or maximal temperature (except C18:0). Finally, mineral composition responded only marginally to the single or combined treatments.

In summary, the prevalence of high temperature along the last stages of fruit maturation was the strongest driving force for shifting most physical and chemical characteristics of green coffee beans. However, these impacts were not so strong as usually reported for field conditions, possibly because our plants were grown under adequate water supply and moderate irradiance. Most importantly, enhanced [CO_2_] could modify and mitigate the heat impact regarding the characteristics related to quality, thus contributing to preserve coffee beans quality closer to that of actual standards. These findings are relevant in the context of predicted global warming scenarios, especially taking into account that previous studies pointed to lower bean quality related to warming. This further highlights that high [CO_2_] will be a key factor for the coffee crop sustainability in face of predicted climate change and global warming scenarios, both regarding the plant (Martins et al., 2016; Rodrigues et al., 2016), the bean, and, therefore, the entire coffee chain of value.

## Authors Contributions

According to their competences, all authors contributed transversally to the several stages of the work, including its design, data acquisition, analysis and interpretation, critically review of the manuscript, and approval of the submitted version. Furthermore, they agree to be accountable for all aspects of the work in ensuring that questions related to the accuracy or integrity of any part of the work were appropriately investigated and resolved.

## Conflict of Interest Statement

The authors declare that the research was conducted in the absence of any commercial or financial relationships that could be construed as a potential conflict of interest.

## Abbreviations

3-CQA, 4-CQA, and 5-CQA: 3-, 4-, and 5-*O*-caffeoylquinic acids
C14:0: myristic acid
C15:0: pentadecylic acid
C16:0: palmitic acid
C16:1: palmitoleic acid
C18:0: stearic acid
C18:1: oleic acid
C18:2: linoleic acid
C18:3: linolenic acid
C20:0: arachidic acid
C20:1: gadoleic acid
C22:0: behenic acid
C24:0: lignoceric acid
CGA: chlorogenic acid
CI: color index
DBI: double bond index
GAE: gallic acid equivalent
H°: Hue angle
TFA: total fatty acids
